# A proceeding to numerical study of mathematical model of bioconvective Maxwell nanofluid flow through a porous stretching surface with nield/convective boundary constraints

**DOI:** 10.1038/s41598-023-48364-2

**Published:** 2024-01-22

**Authors:** Muhammad Imran, Muhammad Abdul Basit, Sumeira Yasmin, Shan Ali Khan, S. K. Elagan, Ali Akgül, Ahmed M. Hassan

**Affiliations:** 1 Department of Mathematics, Government College University Faisalabad, Faisalabad, 38000 Pakistan; 2 Department of Mathematics, College of Science, King Aziz University, P.O. Box 99011, 21955 Jeddah, Saudi Arabia; 3 Department of Computer Science and Mathematics, Lebanese American University, Beirut, Lebanon; 4 Department of Mathematics, Art and Science Faculty, Siirt University, 56100 Siirt, Turkey; 5 Department of Mathematics, Mathematics Research Center, Near East University, Near East Boulevard, 99138 Nicosia, Turkey; 6 Faculty of Engineering, Future University, New Cairo, Egypt

**Keywords:** Applied mathematics, Computational science

## Abstract

Nanofluids become significant in the mass and heat transfer models, especially in engineering problems. Current proceedings focused on the bioconvective Maxwell nanofluid flow passing through the permeable stretchable sheet contingent to nield boundary conditions involving effects of activation energy and thermal radiation. Various physical quantities are involved in this mechanism like magnetic field, thermophoresis, and Brownian motion. The main objective of the study is to report the heat and mass transport in the existence of motile microorganisms. In a mathematical perspective, this structured physical model is going to govern with the help of partial differential equations (PDEs). These governing PDEs are then converted into dimensionless ordinary differential equations form by utilizing appropriate similarity transformations. For numerical results, the shooting technique with ‘bvp4c’ built-in package of MATLAB was implemented. Computed results are then visualized graphically and discussed effects of involving physical variables on the nano-fluid flow profiles are comprehensively. From results, it has been concluded that the fluid flow velocity, temperature, concentration, and microorganism density profiles show escalation on increasing the numeric values of porosity, thermophoresis, buoyancy ratio, bioconvection Rayleigh, Peclet number parameters and decrement reported due to increasing the counts of Prandtl number, magnetic field, radiation, Brownian motion, Lewis number as evident from figures. The numerical outcomes observed by fixing the physical parameters as $0.1 < \lambda < 3.0$, $0.1 < M < 1.5$, $0.1 < Nr < 6.0$, $0.1 < Rb < 1.5$, $0.1 < Nb < 6.0$, $0.1 < Nt < 1.0$, $2.0 < \Pr < 2.9$, $0.1 < Rd < 0.4$. Magnetic field and Brownian motion create retardation impact due to the liquid momentum. In tables, the numerical values of Skin friction, Nusselt number, Sherwood number, and microorganisms density number are presented and also comparison table of our computed results and already published results is included for the validation.

## Introduction

Nanofluid is a suspension or colloidal dispersion consisting of nanoscale solid particles dispersed in a base fluid. The solid particles typically have dimensions in the range of 1 to 100 nm. The base fluid is usually a liquid, such as water, oil, or ethylene glycol, but can also be a gas. The addition of nanoparticles in the base fluid transformed the properties of the base fluid and made it more heat-conductive. It was first introduced by Choi^1^ and utilized in real-world problems. Ahmed et al.^2^ investigated the magnetohydrodynamic (MHD) flow of Maxwell nanoliquid via a stretchable medium having porosity. The study takes into account various additional physical phenomena and boundary conditions, including thermal rays, heat absorption/generation, and convective limiting conditions. Sharma et al.^3^ focused on the numerical and statistical perspective of the study of heat and mass transport through a Maxwell nano-liquid stream passing through a stretchable medium. Salawu et al.^4^ studied thermal transport through a stretchable medium subject to non-linear properties involving Maxwell nanoliquid. Prasannakumara^5^ examined the effect of magnetic dipole over the transport through Maxwell nano-liquid via stretchable sheet numerically. Patil et al.^6^ considered the mutual impacts of thermal rays and chemical processes over the flow behavior of the Maxwell nanofluid. Thermal reactions involve heat generation or absorption within the fluid due to exothermic or endothermic reactions. Chemical reactions refer to the conversion or transformation of species within the fluid, resulting in changes in concentration or properties. Rasool et al.^7^ scrutinized the impacts of Lorentz and Darcy's momentum over the Max-well nanoliquid stream through an isothermally heated stretchable surface.

The fluid flow through the various porous surfaces is of interest in heat and mass transport systems. That was first introduced by the Darcy-Forchheimer law which measures the porosity level of the media. Sajid et al.^8^ investigated Maxwell nanofluid flow through a permeable surface and its permeability is calculated by Darcy-Forchheimer subject to activation energy and thermal radiation. Liu et al.^9^ reported the bioconvective stream of Maxwell nanoliquid through a permeable cylinder under various impacts. Zhou et al.^10^ explored the impacts of temporal rays over the nanoliquid stream through a permeable rotating stretchable disk. Khan et al.^11^ studied a Max-well nano-liquid stream through an infinite vertical plate and additionally included the isothermal and ramped wall concentration and temperature. Al Nuwairan et al.^12^ introduced numerical treatment of heat and mass transport through a Maxwell nanoliquid stream via a porous medium subject to Soret-Dufour influences.

In physical problems, the minimal amount of energy required to initiate a chemical process is known as activation energy. It is required in every mechanism to start a process of heat and mass transport through any type of mechanism. Rashid et al.^13^ used the Darcy-Forchheimer relation to saturate the porous area with the Maxwell fluid. The sheet's exponentially stretchy surface causes flow. At the surface, chemical reaction and Arrhenius energy are taken into consideration. Rafiq et al.^14^ elaborated on the numerous applications of nanofluids that are essential to the success or failure of construction that have resulted from the use of nanofluids and their best considerations. Here, the activation energy and radiation phenomena in the Maxwell nanofluid near the chemically reactive stagnation point have been described in detail. Dessie^15^ investigated the impacts of a minimal amount of energy, chemical process, and temporal rays over the MHD stream of Maxwell liquid through a revolving structure. Rekha et al.^16^ explored how a heat source and sink affected the flow of a nanofluid through a cone, a plate, and a wedge while utilizing a mixture of nano-particles of alloys of alumina (AA7072 and AA7075) in base liquid water. The modeling also takes into account porous material and activation energy. Jayanthi and Niranjan^17^ examined impressions of chemical process, thermal rays, Joule heating, minimal amount of energy, and viscous dissipation on magnetohydrodynamic (MHD) nanofluid stream via a stretched vertical surface.

Thermal radiation plays a vital role in the mass and heat transfer systems which is the inner warmth of the material. During the mechanism of transport, these rays are emitted from the material when particles collide with each other. Madhu et al.^18^ considered the non-Newtonian Maxwell nanofluid boundary layer flow under unstable magnetohydrodynamic (MHD) radiation across a stretched surface. Ramesh et al.^19^ investigated a 3D stream that suffused the permeable area enclosed by the bi-directional stretchable surface having the impact of thermal sink/source and thermal rays. The base liquid is taken into consideration in the Maxwell fluid model. Convective conditions are required in terms of temperature and nanoparticle concentrations. Sreedevi and Reddy^20^ discovered Maxwell nanofluid flows across a stretchable surface under the influence of magnetohydrodynamic (MHD) thermal transport in three dimensions. Mukhtar et al.^21^ developed a mathematical model for boundary layer flow caused by unsteady magnetohydrodynamics (MHD) across a porous stretched surface. Keller-box approach was utilized to analyze the non-Newtonian Maxwell nanofluid under the impact of porous media, thermal radiation, viscous dissipation, and joule heating. Alharbi et al.^22^ by considering heat radiation, looked into the effects of a Maxwell-Sutterby nanofluid on a sheet. The investigation of exponential heat sink/source and minimal energy is presented in this study. Impacts of Brownian motion and thermo-phoresis on motile bacteria are also considered with bioconvection.

In heat and mass transport models heat is transferred by way of convection, when we add gyrotactic motile microorganisms to transfer heat it is termed bioconvection. Hill and Pedley^23^ expressed that the shallow suspensions of randomly distributed, but often upward-swimming microorganisms that are a bit denser than water, bioconvection patterns are typically seen in the laboratory. Alhadhrami et al.^24^ progressed to comprehend the connection between the temperature distribution, magnetic field, and subsequent fluid movement of Maxwell liquid across a stretched sheet. Abdal et al.^25^ analyzed the comparative study of two types of nanofluids, Maxwell and Williamson. In this work, novel concepts such as non-Fourier heat flux, bioconvection of self-moving microorganisms, and activation energy were explored. Khan et al.^26^ discussed the thermal exploration of a Maxwell nanoparticle bioconvective flow across a stretched and rotating cylinder in a porous media. A chemically reactive activation energy comes into touch with the fluid flow. The extending rotary cylinder produces the whirling flow. Acharya^27–32^ evaluated the mass and heat transport through hybrid nano liquid stream through the various interesting geometries like square enclosure, triangular cavity, inclined revolving disk, revolving sphere, and circular cylinder which have many applications in real life and especially in engineering domains. Mohyud-Din et al.^33^ examined the impacts of chemical processes and heat transport over the flow of nanofluid through permeable walls and asymmetric channels. Adnan et al.^34^ investigated the thermal transfer via a nanoliquid stream through the rotating disk. Khan et al.^35,36^ reported the chemical process, soret and dufour impacts over the diverging and converging channels using the Adomain decomposition approach. Secondly, exclaimed by developing the model of a permeable wedge through its nanofluid fluid flow containing motile microorganisms. Ahmed et al.^37^ explained the thermal enhancement characteristics of nanofluid in the applicative frame of reference with dissipative and radiative streams through the wedge. Khan et al.^38^ explored the variations that occurred due to the viscous dissipation and joule heating through the bioconvective stream of nano-liquid in the attendance of microorganisms. Ahmed et al.^39^ discussed the effects of chemical reactions and nonlinear thermal radiation over the hybrid nanoliquid flow through spherical geometry. Das et al.^40^ presented the nanofluid flow through a wedge along with slip conditions and the impact of variable fluid properties over it.

**Figure 1:**
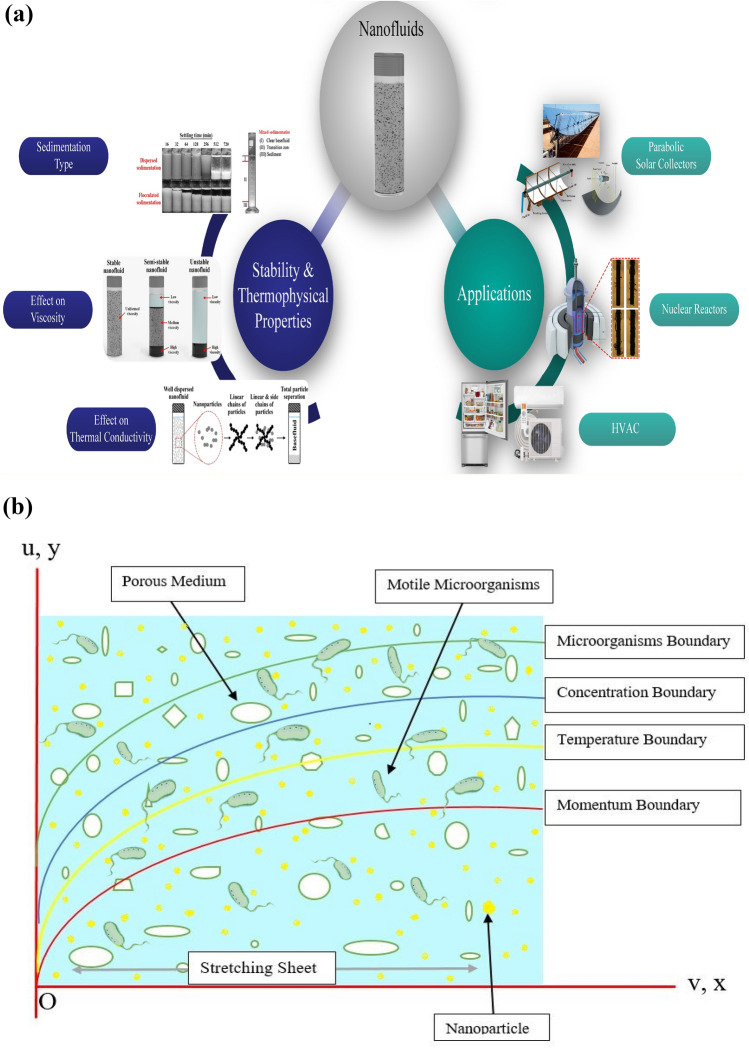
(**a**) Physical utilization of Nanofluids. (**b**) Exhibit the geometry of flow.

The novelty of our work is to evaluate the bio-convective Maxwell nanofluid stream through a stretchable sheet subject to nield/convective boundary constraints in the existence of thermal radiation and minimal energy. This mass and thermal transport model is mathematically developed with the aid of PDE and then these nonlinear PDE’s tackled by implementing a shooting technique along with the bvp4c built-in package of MATLAB. The computed numerical results are represented graphically and the impacts of involving physical quantities on momentum, thermal, concentration, and microorganism depictions are discussed. Stability, thermo-physical properties, and real-world applications of the nanofluid are portrayed in Fig. 1a. The main physical aspects of the present are in the field of industrial, biomedical, and engineering processes. The following questions will be answered in the outcomes section.

- The impact of convective boundary constraints?
- How Darcy Forchheimer and activation energy involvement impact the flow of fluid?
- How Stretching sheet boost the nanofluid flow?

## Mathematical structure

Consider the time-independent two-dimensional electrically charged magneto-hydro-dynamic Darcy-Forchheimer viscous stream of Maxwell nano-liquid under the influence of activation energy through a permeable stretching sheet subject to convective Nield boundary condition as visualized in Fig. 1b. The various impacts like magnetic strength $B_{0}$, and extending rate. The velocity components are taken as u and v as abscissa and ordinate.

Governing equations of the problem are ref^41,42^:

1 $$\frac{\partial u}{{\partial x}} + \frac{\partial v}{{\partial y}} = 0$$

2 $$\begin{aligned} & u\frac{\partial u}{{\partial x}} + v\frac{\partial u}{{\partial y}} + \lambda_{1} \left( {u^{2} \frac{{\partial^{2} u}}{{\partial x^{2} }} + v\frac{{\partial^{2} u}}{{\partial y^{2} }} + 2uv\frac{{\partial^{2} u}}{\partial x\partial y}} \right) = \upsilon \frac{\partial }{\partial y}\left( {\frac{\partial u}{{\partial y}}} \right) \\ & \quad - \frac{{\sigma^{*} B_{0}^{2} }}{{\rho_{f} }}\left( {u + \lambda_{1} v\frac{\partial u}{{\partial y}}} \right) - \frac{\upsilon }{k}u - Fu^{2} \\ & \quad + g\left[ {\frac{1}{{\rho_{f} }}\left\{ {\left( {T - T_{\infty } } \right)\left( {1 - C_{\infty } } \right)\beta \rho_{f} - \left( {C - C_{\infty } } \right)\left( {\rho_{p} - \rho_{f} } \right) - \gamma \left( {N - N_{\infty } } \right)\left( {\rho_{m} - \rho_{f} } \right)} \right\}} \right], \\ \end{aligned}$$

3 $$u\frac{\partial T}{{\partial x}} + v\frac{\partial T}{{\partial y}} = \frac{1}{{\left( {\rho c_{p} } \right)_{f} }}\frac{\partial }{\partial y}\left( {\kappa \frac{\partial T}{{\partial y}}} \right) + \tau \left( {D_{B} \frac{\partial C}{{\partial y}}\frac{\partial T}{{\partial y}} + \frac{{D_{T} }}{{T_{\infty } }}\left( {\frac{\partial T}{{\partial y}}} \right)^{2} } \right) - \frac{1}{{\left( {\rho c} \right)_{f} }}\frac{{\partial q_{r} }}{\partial y},$$

4 $$u\frac{\partial C}{{\partial x}} + v\frac{\partial C}{{\partial y}} = D_{B} \frac{{\partial^{2} C}}{{\partial y^{2} }} + \frac{{D_{T} }}{{T_{\infty } }}\frac{{\partial^{2} T}}{{\partial y^{2} }} - K_{r} \left( {C - C_{\infty } } \right)\left( {\frac{T}{{T_{\infty } }}} \right)^{n} \exp \left( { - \frac{{E_{a} }}{\kappa T}} \right),$$

5 $$u\frac{\partial N}{{\partial x}} + v\frac{\partial N}{{\partial y}} = D_{m} \left( {\frac{{\partial^{2} N}}{{\partial y^{2} }}} \right) - \frac{{bW_{c} }}{{C_{w} - C_{\infty } }}\frac{\partial }{\partial y}\left( {N\frac{\partial C}{{\partial y}}} \right)$$

with

6 $$\begin{aligned} & u = u_{w} \left( x \right) = ax,\,\,v = 0,\,\, - k\frac{\partial T}{{\partial y}} = h_{f} \left( {T_{w} - T} \right),\,\,D_{B} \frac{{\partial^{2} C}}{{\partial y^{2} }} + \frac{{D_{T} }}{{T_{\infty } }}\frac{{\partial^{2} T}}{{\partial y^{2} }} = 0\,\,at\,\,y = 0, \\ & u \to 0,\,\,T \to T_{\infty } ,\,\,C \to C_{\infty } ,\,\,N \to N_{\infty } \,\,as\,\,y \to \infty \\ \end{aligned}$$

where, $q_{r} = \frac{{16a^{*} T_{\infty }^{3} }}{{3k\left( {\rho c} \right)_{f} }}$ be the thermal radiation, $k = k_{\infty } \left( {1 + \varepsilon \frac{{T - T_{\infty } }}{{T_{w} - T_{\infty } }}} \right)$ be the thermal conductivity.

Here, we introduced the similarity quantities for transforming this system of Eqs. (2–6) into ODEs as:

7 $$\begin{aligned} & u = axf^{\prime}\left( \xi \right),\,\,v = - \sqrt {a\upsilon } f\left( \xi \right),\,\,\xi = \sqrt {\frac{a}{\upsilon }} y,\,\, \\ & \theta \left( \xi \right) = \frac{{T - T_{\infty } }}{{T_{w} - T_{\infty } }},\,\,\phi \left( \xi \right) = \frac{{C - C_{\infty } }}{{C_{w} - C_{\infty } }},\,\,\chi \left( \xi \right) = \frac{{N - N_{\infty } }}{{N_{w} - N_{\infty } }} \\ \end{aligned}$$

By implementing similarity variables defined in Eq. (7), we get

8 $$\begin{aligned} & f^{\prime \prime \prime } + \left( {1 + M^{2} F} \right)ff^{\prime \prime } - \left( {\lambda + M} \right)f^{\prime} + F\left( {2ff^{\prime } f^{\prime \prime } - f^{2} f^{\prime \prime \prime } } \right) - \left( {1 + K_{1} } \right)f^{\prime 2} \\ & \quad + \lambda \left( {Nr\phi + \theta + Rb\chi } \right) = 0 \\ \end{aligned}$$

9 $$\begin{aligned} & \left( {\left( {1 + \varepsilon \theta } \right)\frac{4}{3}Rd\left( {1 + \left( {\theta_{w} - 1} \right)\theta } \right)^{3} } \right)\theta^{\prime \prime } + \left( {\varepsilon + 4Rd\left( {\theta_{w} - 1} \right)\left( {1 + \left( {\theta_{w} - 1} \right)\theta } \right)^{2} \theta^{\prime 2} } \right) \\ & \quad + \Pr f\theta^{\prime } + \Pr \left( {Nb\theta^{\prime } \phi^{\prime } + Nt\theta^{\prime 2} } \right) = 0 \\ \end{aligned}$$

10 $$\phi^{\prime \prime } + \Pr Lef\phi^{\prime } + \frac{Nt}{{Nb}}\theta^{\prime \prime } - \Pr Le\sigma \left( {1 + \delta \theta } \right)^{n} \exp \left( { - \frac{E}{1 + \delta \theta }} \right)\phi = 0$$

11 $$\chi^{\prime \prime } + Lbf\chi^{\prime } - Pe\left( {\left( {\chi + \pi } \right)\phi^{\prime \prime } + \chi^{\prime } \phi^{\prime } } \right) = 0$$

With

12 $$\begin{aligned} & f\left( 0 \right) = 0,\,\,f^{\prime } \left( 0 \right) = 1,\,\,\theta \left( 0 \right) = - \gamma \left( {1 - \theta \left( 0 \right)} \right),\,\,Nb\theta^{\prime } \left( 0 \right) + Nt\phi^{\prime } \left( 0 \right) = 0,\,\,\chi \left( 0 \right) = 1\,\,at\,\,\xi = 0, \\ & f^{\prime } \to 0,\,\,\theta \to 0,\,\,\phi \to 0,\,\,\chi \to 0\,\,as\,\,\xi \to \infty \\ \end{aligned}$$

where, the involving physical parameters are

$$\begin{aligned} & F = \lambda_{1} a,\,M^{2} = \frac{{\delta B_{0}^{2} }}{{a\rho_{f} }},\,\lambda = \frac{\upsilon }{Ka},\,K_{1} = \frac{{C_{b} }}{{K^{1/2} }},\,\Pr = \frac{\upsilon }{a},\,Nb = \frac{{\tau D_{B} \left( {C_{w} - C_{\infty } } \right)}}{\upsilon },\,Nt = \frac{{\tau D_{T} \left( {T_{w} - T_{\infty } } \right)}}{{\upsilon T_{\infty } }},\, \\ & Le = \frac{a}{{D_{B} }},\,\delta = \frac{{T_{w} - T_{\infty } }}{{T_{\infty } }},\,E = \frac{{E_{a} }}{{kT_{\infty } }},\,\sigma = \frac{{k_{c}^{2} }}{a},\,Pe = \frac{{bD_{m} W_{c} }}{{D_{B} }},\,Lb = \frac{\upsilon }{{D_{m} }},\,Nr = \frac{{\left( {C_{w} - C_{\infty } } \right)\left( {\rho_{p} - \rho_{f} } \right)}}{{\beta \rho_{f} \left( {T_{w} - T_{\infty } } \right)}}, \\ \end{aligned}$$

Deborah number, Magnetic term, porosity variable, inertial term, Prandtl number, Peclet number, Brownian motion, Bioconvection Lewis number, thermophoresis, Lewis number, temperature difference, Activation energy, reaction rate, Buoyancy ratio parameter respectively.

### Physical quantities

In the perspective of this study the physical quantities involved in every heat and mass transport in the fluid phenomenon, those are required to determine the transport rate of the fluid motion are:

13 $${\text{Skin}}\,{\text{Friction}}\,{\text{number}}\quad C_{f} = \frac{{2\tau_{w} }}{{\rho u_{w}^{2} }},$$

14 $${\text{Nusselt}}\,{\text{number}}\quad Nu_{x} = \frac{{xq_{w} }}{{\kappa \left( {T_{w} - T_{\infty } } \right)}},{\text{where}}\quad q_{w} = - \kappa \left( {\frac{\partial T}{{\partial y}}} \right)_{y = 0} ,$$

15 $${\text{Sherwood}}\,{\text{number}}\,Sh_{x} = \frac{{xq_{m} }}{{D_{B} \left( {C_{w} - C_{\infty } } \right)}},$$

16 $${\text{Microorganism}}\,{\text{density}}\,{\text{number}}\quad {\text{Sn}}_{x} = \frac{{xq_{n} }}{{k\left( {N_{s} - N_{\infty } } \right)}},$$

Dimensionless form of the above quantities is,

17 $$Nu_{x} {\text{Re}}_{x}^{ - 1/2} = - \theta^{\prime } \left( 0 \right),$$

18 $$Sh_{x} {\text{Re}}_{x}^{ - 1/2} = - \phi^{\prime } \left( 0 \right),$$

19 $$Sn_{x} {\text{Re}}_{x}^{ - 1/2} = - \chi^{\prime } \left( 0 \right),$$

And Local Reynolds number is ${\text{Re}}_{x}^{ - 1/2} = xu_{w} /\upsilon$.

## Numerical scheme

The governing system of PDEs (2–6) reduced to dimensionless ODEs (8–11) by implementing similarities introduced in Eq. (7) subject to Nield boundary constraints (12). After transforming the next step is to get numerical solution using the ‘bvp4c’ built-in package of MATLAB. The error bound of this technique is $10^{ - 6}$ which is much lesser as compared to other available techniques which is the reason for the implementation of this technique. The numerical scheme for utilization of this method is as:

20 $$\begin{aligned} & \vartheta_{1} = f,\,\,\vartheta_{2} = f^{\prime } ,\,\,\vartheta_{3} = f^{\prime \prime } ,\,\,\vartheta_{3}^{\prime } = f^{\prime \prime \prime } \\ & \vartheta_{4} = \theta ,\,\,\vartheta_{5} = \theta^{\prime } ,\,\,\vartheta_{5}^{\prime } = \theta^{\prime \prime } \\ & \vartheta_{6} = \phi ,\,\,\vartheta_{7} = \phi^{\prime } ,\,\,\vartheta_{7}^{\prime } = \phi^{\prime \prime } \\ & \vartheta_{8} = \chi ,\,\,\vartheta_{9} = \chi^{\prime } ,\,\,\vartheta_{9}^{\prime } = \chi^{\prime \prime } \\ \end{aligned}$$

21 $$\vartheta_{3}^{\prime } = \frac{1}{{\left( {1 - F\vartheta_{1}^{2} } \right)}}\left[ \begin{gathered} \left( {\lambda + M} \right)\vartheta_{2} - 2F\vartheta_{1} \vartheta_{2} \vartheta_{3} - \left( {1 + M^{2} F} \right)\vartheta_{1} \vartheta_{3} + \left( {1 + K_{1} } \right)\vartheta_{2}^{2} \hfill \\ - \lambda \left( {Nr\vartheta_{6} + \vartheta_{4} + Rb\vartheta_{8} } \right) \hfill \\ \end{gathered} \right]$$

22 $$\vartheta_{5}^{\prime } = \frac{1}{{\left( {\left( {1 + \varepsilon \vartheta_{4} } \right)\frac{4}{3}Rd\left( {1 + \left( {\theta_{w} - 1} \right)\vartheta_{4} } \right)^{3} } \right)}}\left[ \begin{gathered} - \left( {\varepsilon + 4Rd\left( {\theta_{w} - 1} \right)\left( {1 + \left( {\theta_{w} - 1} \right)\vartheta_{4} } \right)^{2} \vartheta_{5}^{2} } \right) \hfill \\ - \Pr \vartheta_{1} \vartheta_{5} + \Pr \left( {Nb\vartheta_{5} \vartheta_{7} + Nt\vartheta_{5}^{2} } \right) \hfill \\ \end{gathered} \right]$$

23 $$\vartheta_{7}^{\prime } = \Pr Le\sigma \left( {1 + \delta \vartheta_{4} } \right)^{n} \exp \left( { - \frac{E}{{1 + \delta \vartheta_{4} }}} \right)\vartheta_{6} - \Pr Lef\vartheta_{7} - \frac{Nt}{{Nb}}\vartheta_{5}^{\prime }$$

24 $$\vartheta_{9}^{\prime } = Pe\left( {\left( {\vartheta_{8} + \pi } \right)\vartheta_{7}^{\prime } + \vartheta_{9} \vartheta_{7} } \right) - Lb\vartheta_{1} \vartheta_{9}$$

## Results and interpretation

**Figure 2:**
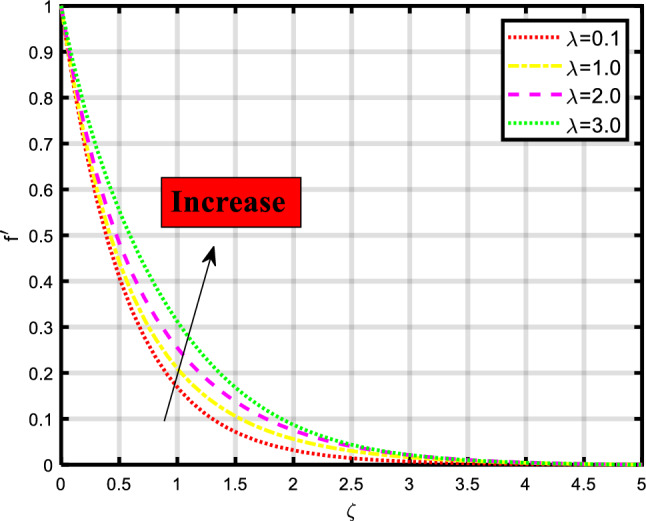
Demonstrated the significance of $\lambda$ over $f^{\prime}$.

**Figure 3:**
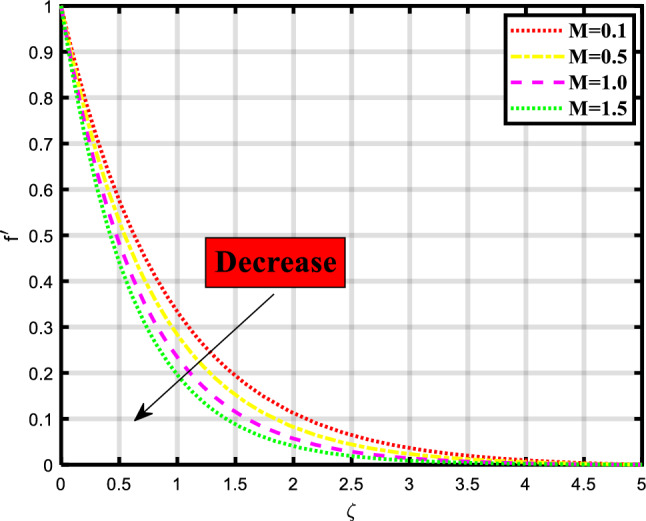
Demonstrated the significance of $M$ over $f^{\prime}$.

**Figure 4:**
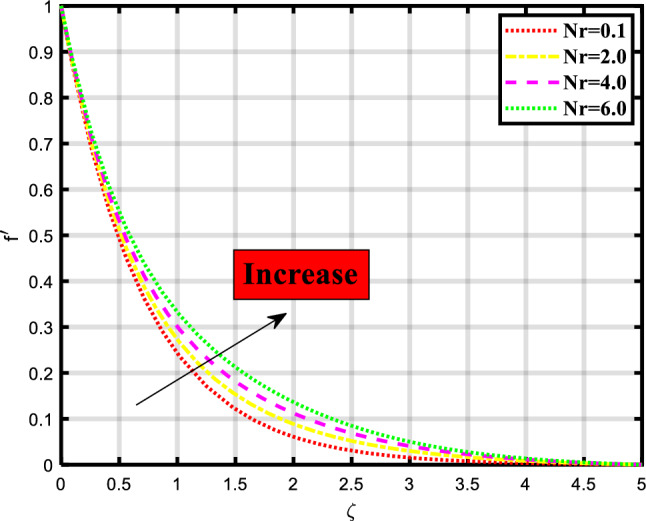
Demonstrated the significance of $Nr$ over $f^{\prime}$.

**Figure 5:**
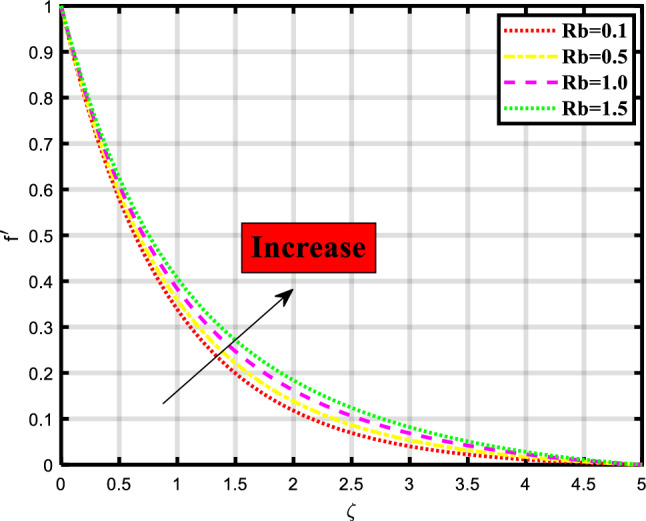
Demonstrated the significance of $Rb$ over $f^{\prime}$.

**Figure 6:**
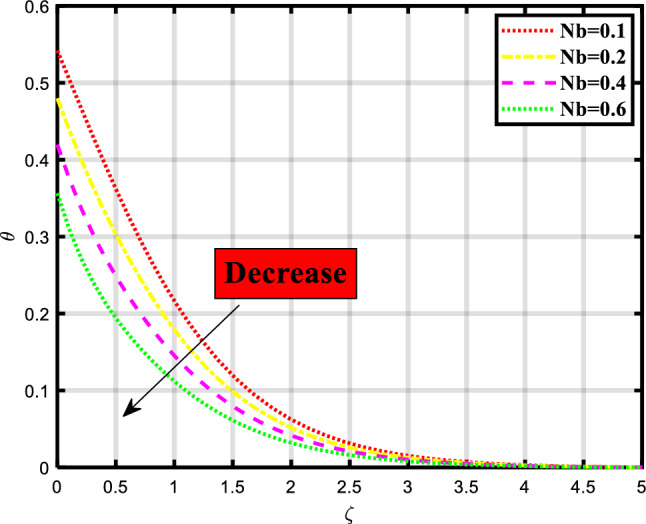
Demonstrated the significance of $Nb$ over $\theta$.

**Figure 7:**
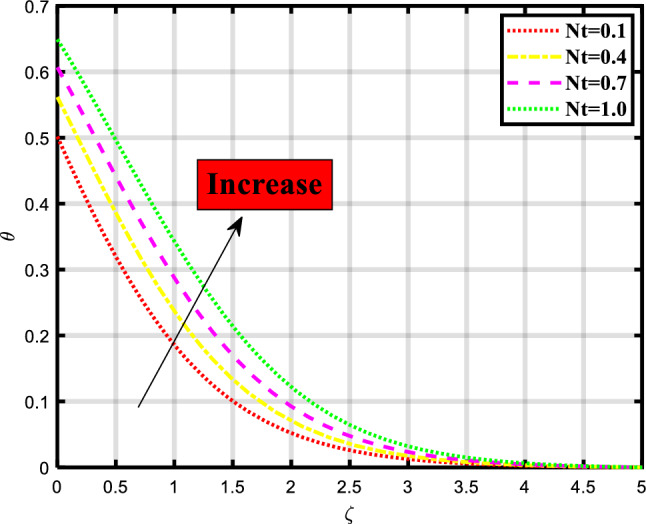
Demonstrated the significance of $Nt$ over $\theta$.

**Figure 8:**
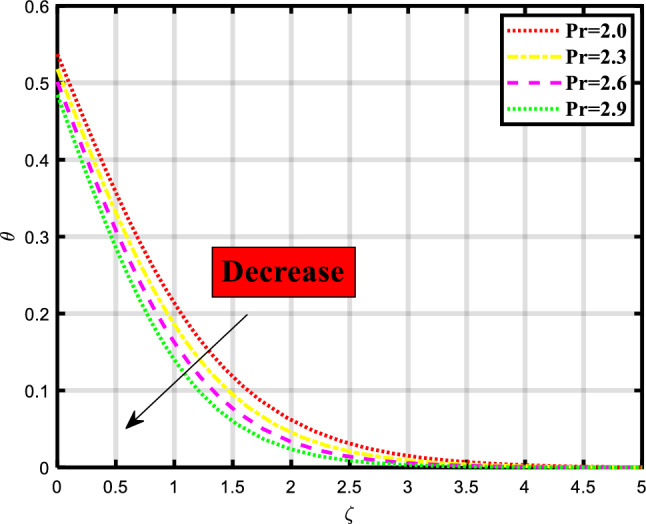
Demonstrated the significance of $\Pr$ over $\theta$.

**Figure 9:**
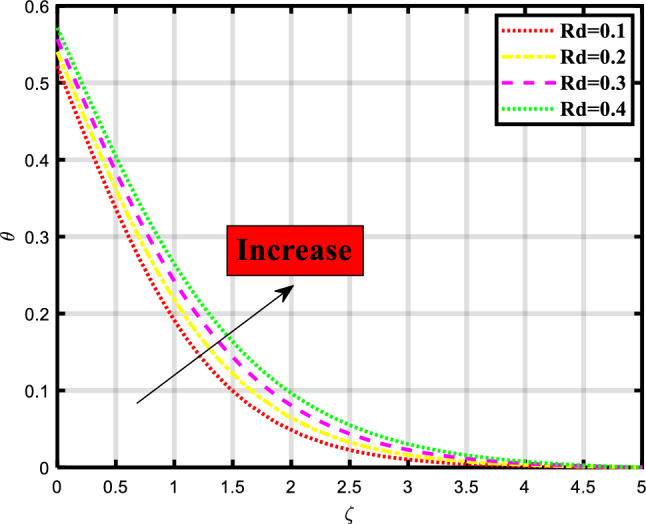
Demonstrated the significance of $Rd$ over $\theta$.

**Figure 10:**
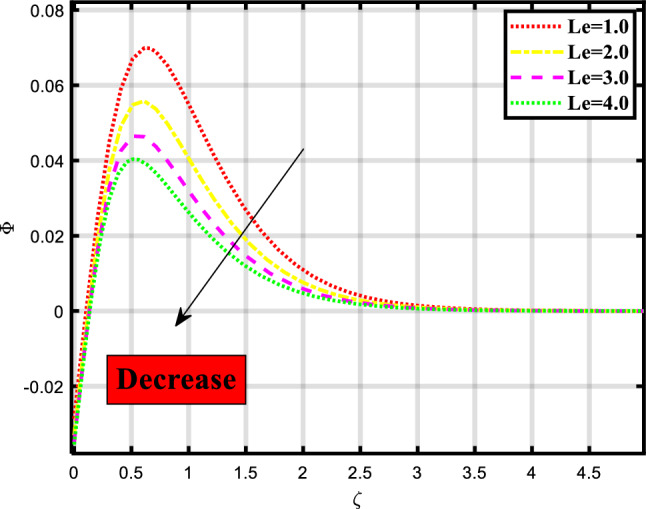
Demonstrated the significance of $Le$ over $\phi$.

**Figure 11:**
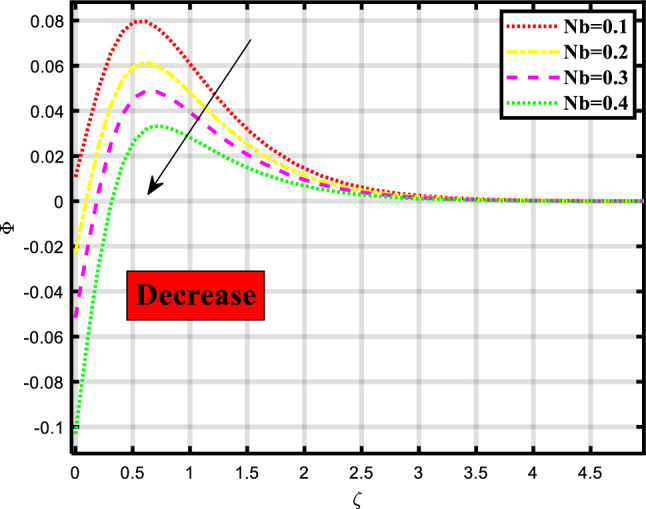
Demonstrated the significance of $Nb$ over $\phi$.

**Figure 12:**
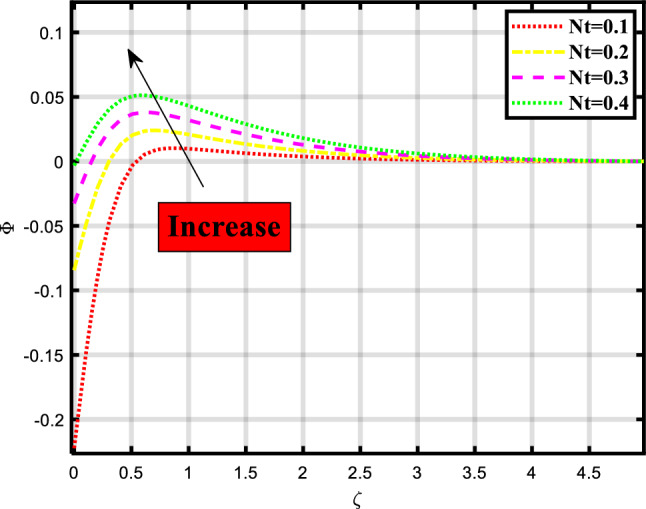
Demonstrated the significance of $Nt$ over $\phi$.

**Figure 13:**
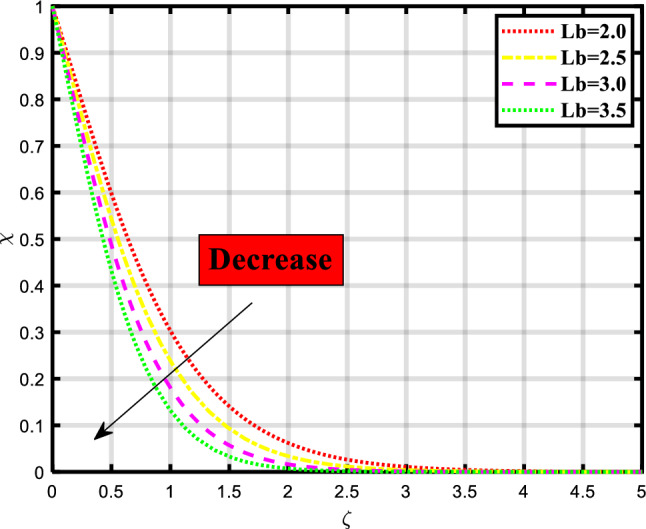
Demonstrated the significance of $Lb$ over $\chi$.

**Figure 14:**
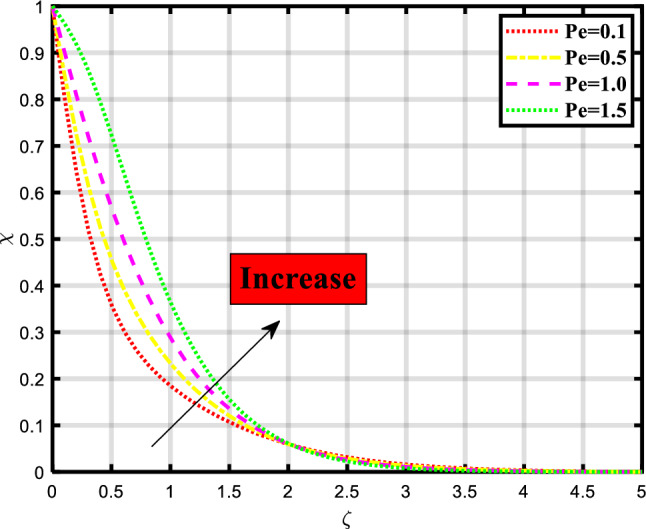
Demonstrated the significance of $Pe$ over $\chi$.

In this section, we discuss and elaborate our proposed and obtained results from the computational analysis of heat transport through the bioconvective flow of Max-well nano-liquid stream through permeable stretchable surface involving impacts of minimal energy, thermal radiation, chemical process, and motile microorganisms subject to nield boundary constraints. This mechanism is mathematical evaluated by constructing the governing system of PDEs and then computationally tackled using the ‘bvp4c’ built-in package of MATLAB. The impacts of various physical quantities are also observed through the graphical visualization of the rate of heat and mass transport. The influence of these physical quantities on the momentum, temperature, concentration, and microorganism profiles is graphically demonstrated in Figs. 2, 3, 4, 5, 6, 7, 8, 9, 10, 11,12, 13 and 14. Some quantities support the fluid flow and several factors minimize the transport ratio. In Fig. 2 impact of the porosity parameter over the velocity profile is demonstrated which shows that by raising the values of the parameter the flow rate also becomes higher. Physically, the intensity ratio of fractures and pores in the material is termed as porosity, and in fluid the volume of interstices in the channel. Figure 3 expounds the significance of the existence of magnetic strength, the increase in parameter shows the decrease in the momentum depiction of the thermal transport problem. Naturally magnetic field attracts the material particles to each other so that’s why the flow of material becomes closer and closer which creates a hurdle to the flow of nanofluid through the sheet. Figure 4 explored the impact of the buoyancy ratio parameter on the velocity profile of the flow model. The flow rate increases when the count of the buoyancy parameter increases. In the stretching/rotating rates, the buoyancy parameter is involved to signify this rate and has importance in such similar mediums. Figure 5 visualizes the influence of bioconvection Rayleigh number in the momentum profile of the flow problem. The flow rate is boosted upon incrementing the values of the variable. Physically, the Rayleigh number is involved when we consider non-Newtonian base fluids and is used to describe the behavior of liquids. Bioconvection Rayleigh number shows the behavior of liquid involving motile microorganisms which shows the density of microbes over the focused region.

The thermal distribution of the flow problem is visualized in Figs. 6, 7, 8 and 9 and the effects of various physical parameters on it are discussed. Figure 6 explores the impact of Brownian motion over the temporal depiction, a downfall in profile occurred while increasing the values of the parameter. Physically, the motion keeps particles from settling down of particles in the suspension and the random motion of particles is considered. The movement of particles depends on the density of the liquid/fluid. Figure 7 envisioned the variation of thermophoresis on the thermal profile of the fluid problem. Undoubtedly due to increasing the values of thermophoresis quantity the temporal profile also booted up. Physically, the difference in how fluid molecules and particle molecules react to temperature gradients gives birth to the thermophoretic force. Due to their size and reduced mobility compared to fluid molecules, particles often have a distinct temperature profile. Because of the concentration gradient that is produced by the temperature gradient around the particles, a net force is applied to them that causes them to move. According to the situation, a quick flow away from the surface is produced by the thermophoretic force caused by a temperature difference. As a result, more hot fluid escapes the surface by flowing off. That leads to a rise in temperature. The temperature is lower when a straight surface is present than when a stretching surface is present, which is a significant finding. Figure 8 expounded the impacts of the Prandtl number on the temporal depiction of heat and mass transport mechanism. The profile depreciated on increasing the count of the Prandtl number. Figure 9 exclaimed the sway of thermal radiation on the temporal profile. the increment in the values of thermal radiation rises the flow of heat and mass transport through a stretching surface. Thermal radiation contributes to the transfer of energy between different parts of a fluid or between a fluid and its surroundings. This can impact the overall energy balance and temperature distribution within the fluid.

The concentration profile of the fluid flow through a stretchable surface depicted in Figs. 10, 11 and 12 is subject to physical parameters. Figure 10 portrays the effect of the Lewis number on the concentration profile of the flow problem. The increment in the Lewis number causes a decrement in the concentration profile. The Lewis number describes how important mass transport and heat transmission are in a fluid. In comparison to the rate at which mass is transported, it measures the rate at which thermal energy is exchanged. Figure 11 shows the manipulation of Brownian motion on the concentration depiction. Brownian motion causes hardship to the smooth flow of heat and mass because when we elevate the numerics of Brownian motion the rate becomes less. Figure 12 exhibits the consequence of thermophoresis on the concentration of microbes depiction, the less the ratio of mass and heat transport the higher the numerics of this parameter.

The density of motile microorganisms depends on the various physical quantities for the transfer of mass and heat through a stretchable medium as shown in Figs. 13 and 14. The effect of bioconvection Lewis number over the microorganism profile which indicates the upsurge in values of parameter causes a downfall in flow rate as evident from Fig. 13. The dynamics of microorganisms or biological particles in response to heat gradients are influenced by the bioconvection Lewis number. With a low Lb, the thermal diffusivity is more dominant than the mass diffusivity, which causes intense particle motion and aggregation in hotter areas. A high Lb, on the other hand, suggests that the mass diffusivity is more substantial, leading to less noticeable particle motion and aggregation. Figure 14 explored the variation caused due to the presence of Peclet number in the microorganism profile. An increase in flow rate occurred while rising the count of parameters. The effectiveness and rate of transfer are influenced by the Peclet number. Convection has a major role when the Peclet number is large, leading to fast movement and increased rates of mass or heat transfer. A high Peclet number, on the other hand, denotes that diffusion is the main mechanism of transport, resulting in slower transfer rates.

## Tabular analysis

In Tables 1, 2, 3 and 4 the numerical behavior of physical quantities are elaborated, from the numeric values of tables local skin friction, Nusselt number, Sherwood number, and microorganisms density number show upsurge and downfall by variating the values of involving various parameters and fixing the values as $0.2 \le \lambda \le 0.6$, $0.2 \le M \le 0.4$, $0.2 \le Nr \le 0.4$, $0.2 \le Rb \le 0.6$, $2.0 \le Le \le 4.0$, $0.2 \le Lb \le 0.6$, $3.0 \le Pe \le 5.0$. Table 5 certifies that at fixed values of M, our reported results are very much in accord with the previously published results. The finest outcomes received through this mechanism impacted to highly on the heat and mass transport system.

**Table 1:** Shows contrast of $\lambda ,\,M,\,Nr,$ and, $Rb$ on the Local skin friction factor.

$\lambda$	$M$	$Nr$	$Rb$	$- f^{\prime\prime}(0)$
0.2	0.1	0.1	0.1	1.0987
0.4				1.1049
0.6				1.1124
0.1	0.2	0.1	0.1	1.1401
	0.3			1.1828
	0.4			1.2243
0.1	0.1	0.2	0.1	1.0959
		0.3		1.0956
		0.4		1.0953
0.1	0.1	0.1	0.2	1.0920
			0.4	1.0836
			0.6	1.0752

**Table 2:** Shows contrast of $Nb,\,Nt,\,\Pr ,$ and, $Rd$ on local Nusselt number.

$Nb$	$Nt$	$\Pr$	$Rd$	$- \theta^{\prime}(0)$
0.4	0.3	0.2	0.1	0.3038
0.6				0.3195
0.8				0.3445
0.2	0.4	0.2	0.1	0.2898
	0.5			0.2847
	0.6			0.2798
0.2	0.3	0.3	0.1	0.2027
		0.4		0.2158
		0.5		0.2282
0.2	0.3	0.2	0.2	0.1888
			0.4	0.1889
			0.6	0.1890

**Table 3:** Shows contrast of $Le,\,Nb,$ and, $Nt$ on local Sherwood number.

$Le$	$Nb$	$Nt$	$- \phi^{\prime}(0)$
2.0	0.1	0.2	1.3328
3.0			2.6596
4.0			4.1051
1.0	0.2	0.2	2.0049
	0.3		2.0067
	0.4		2.0124
1.0	0.1	0.3	2.0147
		0.4	2.0180
		0.5	2.0198

**Table 4:** Shows contrast of $Lb$ and $Pe$ on local microorganism density number.

$Lb$	$Pe$	$Nc$	$- \chi^{\prime}(0)$
0.2	2.0	0.1	0.2415
0.4			0.3193
0.6			0.3984
0.1	3.0	0.1	0.2017
	4.0		0.2005
	5.0		0.2000
0.1	2.0	0.2	0.1947
		0.4	0.1985
		0.6	0.2001

**Table 5:** Shows co-relation with previously published results with our computed numerics for *M* = 0.0, 0.5, 0.8, 1.0.

M	Muhammad et al.^43^	Jawad et al.^41^	Current results
0.0	0.20177	0.20178	0.20176
0.5	0.19952	0.19954	0.19955
0.8	0.19630	0.19633	0.19633
1.0	0.19331	0.19331	0.19331

## Conclusion

In the study of bioconvective Maxwell nano-liquid stream over a permeable stretchable surface subject to nield boundary conditions and impacts of various physical quantities like activation energy, thermal radiation, and chemical process. The problem is mathematically solved by developing governing PDEs and then numerically tackled using the ‘bvp4c’ built-in package of MATLAB. The notable outcomes are:

- On enhancing the magnetic characteristic and Brownian motion the liquid momentum profile lowers owing to happening of retardation impact.
- As buoyancy parameter's values increase, the fluid's motion increases as well, which causes the velocity profiles to increase.
- By enlarging the counts of Nb, the concentration and thermal profiles show decrement in the transport of mass and heat.
- There is a reduction in the mass flow that causes the concentration profile to deteriorate for an increase in the Lewis and Brownian numbers.
- The profile of microorganisms is inclined by an increase in the Peclet number, which causes a decrease in the diffusivity of motile microbes.

In future this approach may be implemented using some other available heat and mass flux models and also by geometrical change.

## Data Availability

The datasets used and/or analyzed during the current study are available in the manuscript. Any additional information or data required available from corresponding author upon reasonable request.

## List of symbols

$$x,y$$: Cartesian coordinates (–)
$D_{m} \,$: Microorganism’s diffusion coefficient (–)
$$u,v$$: Velocity components (m/s)
$$\Pr$$: Prandtl number (–)
$k$: Nanofluid thermal conductivity (W/m K)
$E$: Activation energy variable (–)
$C_{f}$: Skin friction parameter (–)
$C_{\infty }$: Free stream concentration (mol/m^3^)
$q_{r}$: Radiative heat flux (W/m^2^)
$$T_{\infty }$$: Ambient temperature (T)
$D_{B}$: Brownian motion coefficient (m^2^/s)
$$N$$: Microorganism’s density (–)
$B_{0}$: Magnetic parameter (Telsa)
$$N_{w}$$: Surface microorganisms (–)
$C$: Concentration of particles (mol/m^3^)
$Nb$: Brownian motion parameter (–)
$$Sc$$: Schmidt number (–)
$Nc$: Bioconvection Rayleigh number (–)
$$k_{c}$$: Chemical reaction (–)
$T$: Temperature of particles (K)
$$M$$: Magnetic strength (–)
$\left( {\rho C_{p} } \right)_{nf}$: Heat capacity of nanofluid (J/kg K)
$$Pe$$: Peclet number (–)
$$D_{T}$$: Thermophoresis coefficient (m^2^/s)
$C_{w}$: Surface concentration (mol/m^3^)
$$T_{w}$$: Surface temperature (T)
$$N_{\infty }$$: Ambient microorganisms (–)
$$Nt$$: Thermophoresis parameter (–)
$Nr$: Buoyancy ratio parameter (–)
$$Rd$$: Thermal radiation (–)
$Le$: Bioconvection Lewis number (–)

## Greek symbols

$\rho_{p}$: Nanoparticle density (kg/m^3^)
$\mu$: Dynamic viscosity (kg/m s)
$\sigma$: Stefan-Boltzmann constant (–)
$$\alpha$$: Thermal diffusivity (m^2^/s)
$\tau$: Ratio of heat capacitance (–)
$\delta$: Thermal ratio parameter (–)
$$\beta$$: Volume suspension (–)
$\lambda$: Mixed convection parameter (–)
$$\upsilon$$: Kinematic viscosity (m^2^/s)
$\gamma$: Volume fraction of microorganisms (–)
$\rho_{f}$: Nanofluid density (kg/m^3^)
